# Impact of Pre‐Treatment Serum Ferritin on Response and Survival in Myelodysplastic Syndromes Treated With Azacytidine: A Multivariate Analysis

**DOI:** 10.1002/cam4.71127

**Published:** 2025-08-04

**Authors:** Gloria Moreno Carrasco, Rodolfo Matias Ortiz Flores, Regina García Delgado, Borja Cidoncha Morcillo, Ana Isabel Rosell Mas, Dana Díaz Canales, María Rodríguez González, Manuel Carrasco Gomariz, Alejandro Escamilla‐Sánchez

**Affiliations:** ^1^ UGC Hematología y Hemoterapia Hospital Universitario Virgen de la Victoria Málaga Spain; ^2^ BE21‐Hematología e Inmunoterapia Instituto de Investigación Biomédica de Málaga y Plataforma en Nanomedicina (IBIMA Plataforma BIONAND) Málaga Spain; ^3^ Departamento de Fisiología Humana, Histología Humana, Anatomía Patológica y Educación Físico‐Deportiva, Facultad de Medicina Universidad de Málaga Málaga Spain; ^4^ UGC Farmacia Hospital Universitario Virgen de la Victoria Málaga Spain

**Keywords:** azacytidine, ferritin, hypomethylating agents, iron overload, myelodysplastic syndrome, overall survival, prognostic biomarker, retrospective cohort study, transfusion dependency, treatment response

## Abstract

**Background:**

Myelodysplastic syndromes (MDS) encompass a heterogeneous group of haematological neoplasms characterized by ineffective haematopoiesis and a variable risk of transformation to acute myeloid leukaemia (AML). Elevated serum ferritin (SF), a marker of iron overload (IO), has been linked to poorer outcomes in MDS. However, the impact of pre‐treatment SF levels on azacytidine (AZA) response and survival outcomes remains unclear.

**Methods:**

This retrospective cohort study included patients with World Health Organization‐defined MDS or AML with 20%–30% bone marrow blasts treated with AZA at the Virgen de la Victoria University Hospital (Málaga, Spain) from 2007 onwards. Patients were stratified into three groups based on pre‐treatment SF levels: < 500 ng/mL, 500–1000 ng/mL and > 1000 ng/mL. Logistic regression and Kaplan–Meier methods were used to analyse overall response (OR) and overall survival (OS).

**Results:**

Among 240 patients, 190 with available SF data were analysed. Patients with SF > 1000 ng/mL showed significantly lower OR (24.2%) and shorter OS (median: 10.1 months) compared to those with SF < 500 ng/mL (OR: 71.4%, OS: 18.2 months) and 500–1000 ng/mL (OR: 82.6%, OS: 20.5 months) (*p* < 0.0001 for OR, *p* = 0.001 for OS). Multivariate analysis confirmed elevated SF as an independent predictor of poorer outcomes.

**Conclusions:**

Elevated pre‐treatment SF levels are strongly associated with reduced response and survival in patients with MDS or AML treated with AZA. Early IO management, such as iron chelation, may improve treatment outcomes.

## Graphical abstract

Elevated serum ferritin levels before treatment with azacytidine are associated with lower response rates and shorter survival in patients with myelodysplastic syndromes. This study supports the early identification of transfusion‐related iron overload to improve treatment planning.

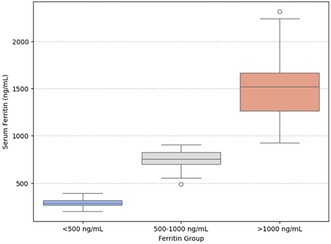

## 1 Introduction

Myelodysplastic syndromes (MDS), as classified by the French‐American‐British (FAB) system including diseases such as refractory anaemia and chronic myelomonocytic leukaemia, represent a diverse spectrum of haematological neoplasms marked by ineffective haematopoiesis and a highly variable risk of transformation to acute myeloid leukaemia (AML) [1]. This disease heterogeneity encompasses a wide range of cytogenetic anomalies and impaired differentiation of haematopoietic progenitors, contributing to variable clinical outcomes [2]. Among patients with MDS, dependence on red blood cell (RBC) transfusions and iron overload (IO) has emerged as critical prognostic indicators, adversely influencing both OS and quality of life [3, 4, 5].

Serum ferritin (SF), an established surrogate marker of iron stores, is commonly employed to evaluate IO in patients with MDS. Elevated SF levels correlate with an increased risk of organ dysfunction, including cardiac, hepatic and endocrine complications, thereby contributing to poorer clinical outcomes [6]. Furthermore, IO is associated with oxidative stress and cellular injury, which can potentially diminish the efficacy of therapies such as azacytidine (AZA) [7, 8]. Patients with MDS, particularly those classified as low‐risk, often rely on transfusional support to maintain adequate haemoglobin levels and improve quality of life. However, repeated transfusions can lead to secondary IO, which may interfere with the efficacy of subsequent treatments, including hypomethylating agents. In the present study, we evaluated a cohort of patients with transfusion‐related IO, using the FAB classification system as recorded in their clinical documentation at the time of diagnosis. Due to incomplete cytogenetic data in some cases and the retrospective nature of this work, no reclassification was performed, as it was not essential to address the primary objective of this analysis.

AZA has demonstrated efficacy in improving haematopoiesis, reducing transfusion dependency and extending OS in MDS [9]. Its mechanisms include epigenetic modulation, as shown by Thoms et al., who reported CpG methylation changes in haematopoietic progenitors, and Raj et al., who observed gene‐specific demethylation in responsive patients [10, 11]. Clinically, AZA at 75 mg/m^2^ for 7 days every 28 days achieves haematological response rates of 37.5%–49% in high‐risk MDS [12]. It enhances erythroid progenitor function, increasing CFU‐e and BFU‐e colony formation, which correlates with higher haemoglobin levels and transfusion independence [13]. Additionally, improved responses have been linked to STAT3/STAT5 pathway modulation and enhanced megakaryocytic differentiation [11, 12]. Despite these benefits, the impact of pre‐treatment SF levels on response and survival remains unclear. This study explores whether elevated SF predicts worse outcomes in MDS patients treated with AZA.

Recent prognostic models have incorporated pre‐transplant SF levels as a predictor of survival following stem cell transplantation (SCT) for acute leukaemia or MDS [9]. These findings, coupled with the results presented in this study, underscore the prognostic utility of measuring pre‐SCT SF levels to predict transplantation outcomes in patients with AML or MDS, particularly in the setting of advanced MDS and high‐risk AML, where elevated SF has been identified as an unfavourable factor for survival post‐allogeneic SCT [14, 15]. Emerging evidence further suggests that IO is an important predictor of SCT outcomes [9].

The aim of this work was to address the impact of pre‐treatment SF levels on OR and OS in patients with World Health Organization (WHO)‐defined MDS or AML with 20%–30% bone marrow blasts treated with AZA through a compassionate‐use program in Spain. We hypothesise that elevated pre‐treatment SF levels are associated with suboptimal response rates and inferior OS, emphasising the need for timely interventions to mitigate iron accumulation in this patient population.

## 2 Methods

### 2.1 Study Design and Patient Population

This observational retrospective cohort study aims to assess the impact of pre‐treatment SF levels on OR and OS in patients with MDS or AML with 20%–30% bone marrow blasts treated with AZA. Eligible patients were diagnosed between 2007 and 2023 and met WHO criteria for MDS or AML with 20%–30% bone marrow blasts, consistent with the diagnostic standards in use during the early years of the study period and received AZA as part of their treatment regimen.

### 2.2 Data Collection

Patient demographics, clinical characteristics and laboratory data were retrospectively extracted from medical records. Baseline variables included age, sex, bone marrow blast percentage, International Prognostic Scoring System (IPSS) risk category (selected as it was the accepted standard during the primary data collection period), haemoglobin level, platelet count, absolute neutrophil count, transfusion dependency and pre‐treatment SF levels. Although more recent prognostic models such as IPSS‐R and IPSS‐M have been developed, the original IPSS classification was used in this study because it was the validated, sufficient and clinically accepted standard at the time of diagnosis and therapeutic decision‐making for the majority of patients. This framework provided the necessary prognostic stratification according to prevailing guidelines during the data collection period. Retrospective reclassification was not feasible due to the lack of comprehensive molecular data. Patients were categorised into three groups based on pre‐treatment SF levels: < 500 ng/mL, 500–1000 ng/mL and > 1000 ng/mL. The thresholds were determined based on prior studies, which indicate that SF levels exceeding 1000 ng/mL are significantly associated with an increased risk of IO‐related complications, while 500 ng/mL serves as a reference point distinguishing normal from moderately elevated iron stores [3, 9]. No patients received erythropoiesis‐stimulating agents (ESAs) prior to AZA initiation, as all belonged to intermediate‐2 or high IPSS risk categories, where ESA use is not routinely indicated.

### 2.3 Treatment and Response Assessment

All patients received AZA at a dose of 75 mg/m^2^ administered subcutaneously for 7 consecutive days every 28 days, following standard clinical practice guidelines. Haematological responses were assessed based on the International Working Group (IWG) criteria for MDS (2006) and AML (2003), as these were the accepted standards during the period of data collection. Responses were classified into the following categories: complete response (CR), marrow complete response (mCR), partial response (PR), haematological improvement (HI), stable disease (SD) or progressive disease (PD). The best overall response (BOR) included CR, mCR, PR, HI and complete cytogenetic response (CCyR).

### 2.4 Statistical Analysis

Baseline characteristics across SF level groups were compared using chi‐squared, Fisher's exact test or likelihood ratio chi‐square for categorical variables, and Mann–Whitney *U*, Wilcoxon rank‐sum or Kruskal–Wallis tests for continuous variables. Logistic regression was utilised to evaluate the impact of pre‐treatment SF levels on OR, adjusting for potential confounders, including age, sex, IPSS risk category and transfusion dependence. OS was evaluated using the Kaplan–Meier method, with group comparisons performed using the log‐rank test. Independent prognostic factors for OS were identified using a Cox proportional hazards model. The proportional hazards assumption was tested using Schoenfeld residuals. The test revealed that ferritin did not meet the proportional hazards assumption (*p* = 0.0083), while all other covariates were proportional (*p* > 0.05). To address this, a stratified Cox model was applied. Given the retrospective design and moderate sample size of our cohort, more advanced time‐varying models were not applied, as they could compromise statistical robustness and increase the risk of overfitting. The stratified Cox model was considered the most appropriate method to account for non‐proportional hazards in this context. SF was selected as the primary variable in the multivariate model because it is a well‐established surrogate marker for IO, integrating both transfusion‐dependent and transfusion‐independent iron accumulation. Prior studies have demonstrated its prognostic impact on survival and treatment response, beyond transfusion history alone [3, 10]. Additionally, univariate analysis showed that while transfusion dependency was associated with OS, its effect was largely mediated by ferritin levels, which remained independently significant in multivariate analysis. As explained above, no reclassification of MDS subtypes was performed, since ferritin levels were the primary prognostic variable of interest [7]. All statistical analyses were conducted using SAS software (version 9.2; SAS Institute, Cary, NC, USA). Data with *p* < 0.05 were considered statistically significant.

### 2.5 Ethical Considerations

The study was conducted in accordance with the Declaration of Helsinki and was approved by the Ethics Committee of the Hospital Universitario Virgen de la Victoria, Málaga, Spain. Informed consent was obtained from all patients prior to their inclusion in the compassionate‐use program, following all applicable ethical regulations.

## 3 Results

### 3.1 Univariate and Multivariate Analysis

Table 1 presents the univariate and multivariate analysis results for OS. Pre‐treatment SF levels > 1000 ng/mL remained an independent predictor of shorter OS (adjusted HR: 1.85, 95% CI: 1.32–2.61, *p* = 0.0012), even after adjusting for IPSS, transfusion dependency and age.

**TABLE 1:** Univariate and multivariate analysis results for overall survival (OS).

	Variable	Univariate HR (95% CI)	Univariate *p* value	Multivariate HR (95% CI)	Multivariate *p* value
0	Ferritin > 1000 ng/mL	2.10 (1.50–2.95)	0.0001	1.85 (1.32–2.61)	0.0012
1	Age	1.05 (1.01–1.09)	0.0050	1.04 (1.00–1.08)	0.0150
2	IPSS high	1.75 (1.22–2.51)	0.0020	1.42 (1.05–2.08)	0.0280
3	Transfusion dependency	1.98 (1.45–2.72)	0.0003	1.72 (1.30–2.35)	0.0025

### 3.2 Baseline Characteristics of the Study Population

**FIGURE 1:**
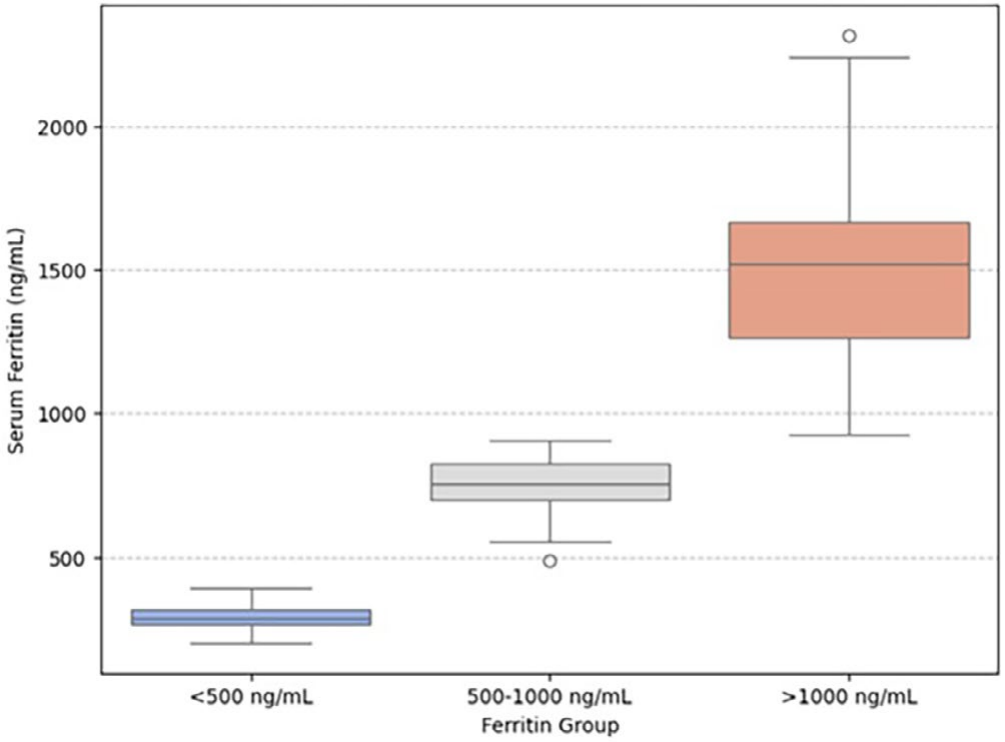
Boxplot of pre‐treatment serum ferritin levels stratified into three groups: < 500 ng/mL, 500–1000 ng/mL and > 1000 ng/mL. The plot illustrates the distribution, median and variability of ferritin levels in each category, providing a clearer representation of data dispersion compared to histograms.

The study cohort included 240 patients treated at our hospital. Of these, 190 patients had baseline and post‐AZA SF data available, meeting the inclusion criteria for this analysis. The majority of patients were male (*n* = 160; 66.9%), with a median age of 70 years (range, 28–86 years). Information on prior treatments, including ESAs such as erythropoietin or luspatercept, as well as the number of transfusions before AZA initiation, was not systematically recorded in the available dataset. Therefore, these potential confounders could not be included in the analysis. The median time from diagnosis to the initiation of AZA treatment was 205 days (95% CI, 61–671 days). Diagnoses in the overall cohort were categorised according to the FAB system: 90 patients (41.8%) had refractory anaemia with excess blasts (RAEB), 59 (27.4%) had refractory anaemia (RA), 32 (14.9%) had refractory anaemia with ringed sideroblasts (RARS), 18 (8.4%) had chronic myelomonocytic leukaemia (CMML) and 16 (7.4%) had RAEB in transformation (RAEB‐t) (Table 2). IPSS risk stratification of the patients identified 24 (10.2%) as high‐risk, 46 (19.6%) as low‐risk, 18 (7.6%) as unknown or non‐valuable, 92 (39.1%) as intermediate‐1 (Int‐1) and 55 (23.4%) as intermediate‐2 (Int‐2) patients (Table 2). Among the 190 patients with evaluable SF levels, the mean pre‐AZA ferritin level was 1286.3 ng/mL, while the median ferritin level was 1001 ng/mL (Table 3). In Figure 1, the distribution of pre‐treatment SF levels across the three study groups is shown in the boxplot highlights. Median, interquartile range (IQR) and outliers provide a more detailed visualisation of data variability.

**TABLE 2:** Patient baseline characteristics according to pre‐AZA SF levels.

Characteristic	SF < 500 ng/mL (*n* = 49)	SF 500–1000 ng/mL (*n* = 46)	SF > 1000 ng/mL (*n* = 95)	Total (*N* = 190)	*p*
Median age, years	69	70	70	70	0.9788
Male, %	75.5	65.2	69.2	69.8	0.5390
IPSS risk group, %					
Low	28.6	24.4	14.1	20.4	0.5653
Int‐1	34.7	33.3	41.3	37.6	
Int‐2	22.5	24.4	26.1	24.7	
High	8.2	13.3	8.7	9.7	
Unknown	6.1	4.4	9.8	7.5	
Absolute neutrophil count, mean ± SD, g/L	2.0 ± 2.2	2.3 ± 2.1	3.0 ± 8.6	2.6 ± 6.3	0.6285
Platelets, mean ± SD, g/L	140.1 ± 127.2	167.5 ± 155.9	146.7 ± 161.4	149.9 ± 151.4	0.6601
Hb level, mean ± SD, g/dL	10.3 ± 2.1	9.0 ± 1.7	9.5 ± 1.9	9.6 ± 1.9	0.0041
RBC‐transfusion dependence, %	65.3	87.0	80.0	77.9	0.0362

Abbreviations: Int, intermediate; SD, standard deviation.

**TABLE 3:** Pre‐AZA ferritin levels.

*N*	Mean	Median	Std dev	Minimum	Maximum	Lower quartile	Upper quartile	*N* miss
190	1286.300	1001.000	1039.534	21.000	5548.000	480.000	1746.000	50

### 3.3 Overall Response

The 190 patients with available pre‐AZA SF levels were divided into three groups: < 500 ng/mL SF (*n* = 49), 500–1000 ng/mL SF (*n* = 46) and > 1000 ng/mL SF (*n* = 95). The overall response rate (ORR) varied significantly across the SF level groups. Patients with SF levels < 500 ng/mL and 500–1000 ng/mL demonstrated higher ORR compared to those with SF > 1000 ng/mL. Specifically, ORR was 71.4% in the < 500 ng/mL group, 82.6% in the 500–1000 ng/mL group and 24.2% in the > 1000 ng/mL group (*p* < 0.0001) (Table 4). Logistic regression analysis revealed that pre‐treatment SF levels > 1000 ng/mL were significantly associated with a lower likelihood of achieving OR compared to SF levels < 500 ng/mL (adjusted OR = 0.23, 95% CI: 0.11–0.49, *p* < 0.0001). Additionally, treatment responses varied substantially across IPSS risk categories. As shown in Table S1, ORRs were highest in low‐risk patients (62.5%), followed by intermediate‐1 (48.9%) and intermediate‐2 (45.4%) categories. In contrast, no major responses were observed among patients classified as high‐risk (0.0%). This trend persisted at 4 and 6 months, with gradual increases in response over time in intermediate‐risk groups. To better understand the impact of disease risk on treatment outcomes, ORRs were also stratified according to the IPSS risk categories. Full response data stratified by IPSS risk categories are provided in Table S1. Patients with low‐risk MDS had the highest OR (62.5%), whereas high‐risk MDS showed no significant responses. A similar trend was observed at 4 and 6 months, with response rates increasing over time in intermediate‐risk groups.

**TABLE 4:** Best response according to SF levels.

Characteristic, %	SF < 500 ng/mL (*n* = 49)	SF 500–1000 ng/mL (*n* = 46)	SF > 1000 ng/mL (*n* = 95)	*p*
OR	71.4	82.6	24.2	< 0.0001
CR	30.6	41.3	3.2	< 0.0001
mCR	24.5	15.2	6.3	0.0083
PR	12.3	13.0	5.3	0.2035
HI	4.1	13.0	9.5	0.3006
Stable disease	8.2	17.4	24.2	0.0613
Progressive disease	18.4	0	50.5	< 0.0001
Missing	2.0	0	1.1	0.6222

*Note:* Statistical comparisons between ferritin groups were performed using Fisher's exact test. Values represent two‐sided *p* values.

### 3.4 Survival Outcomes

**FIGURE 2:**
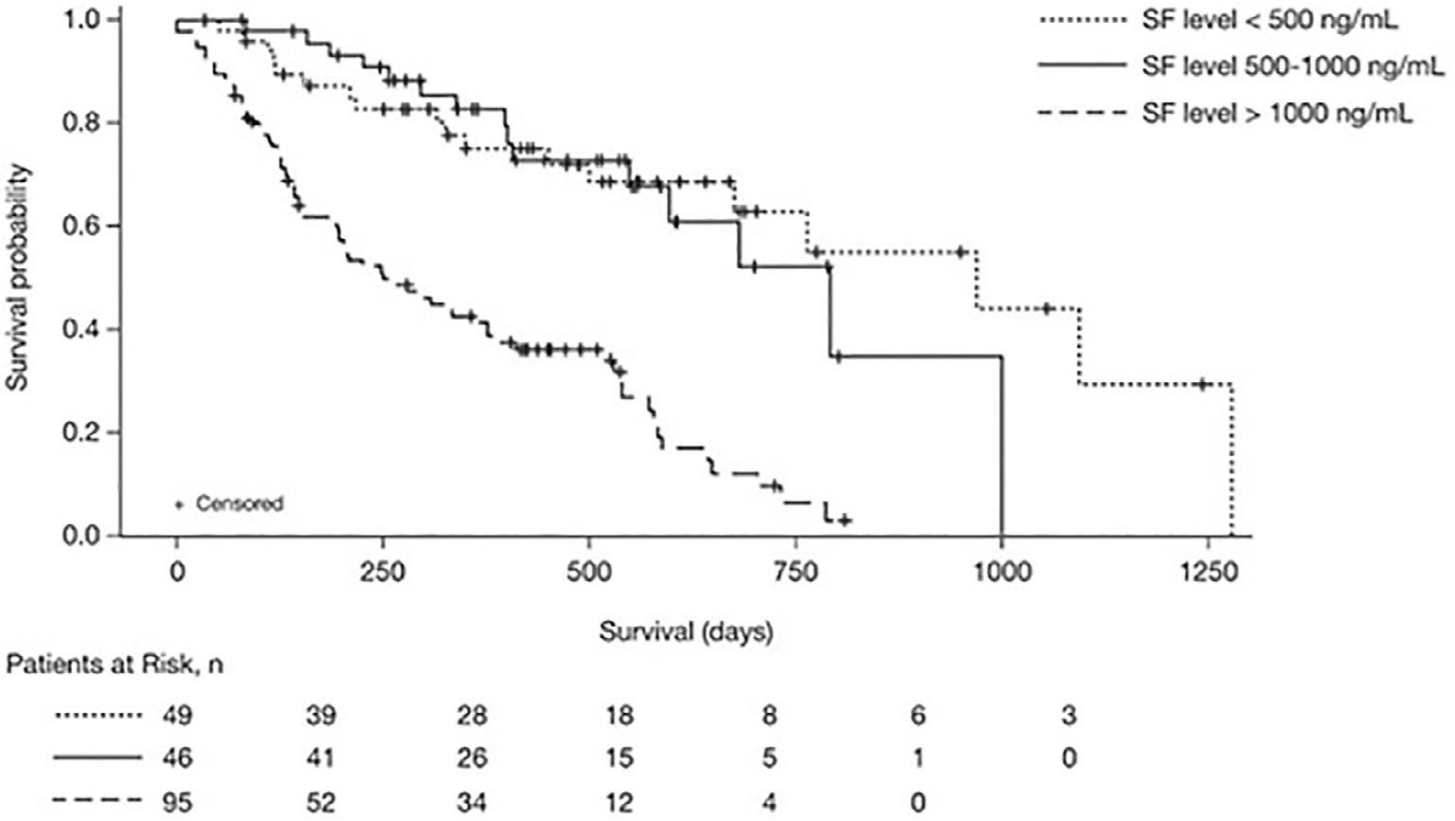
Schoenfeld residuals for ferritin levels in the Cox proportional hazards model. The non‐constant trend over time suggests a violation of the proportional hazards assumption (*p* = 0.0083), requiring a stratified Cox model.

**FIGURE 3:**
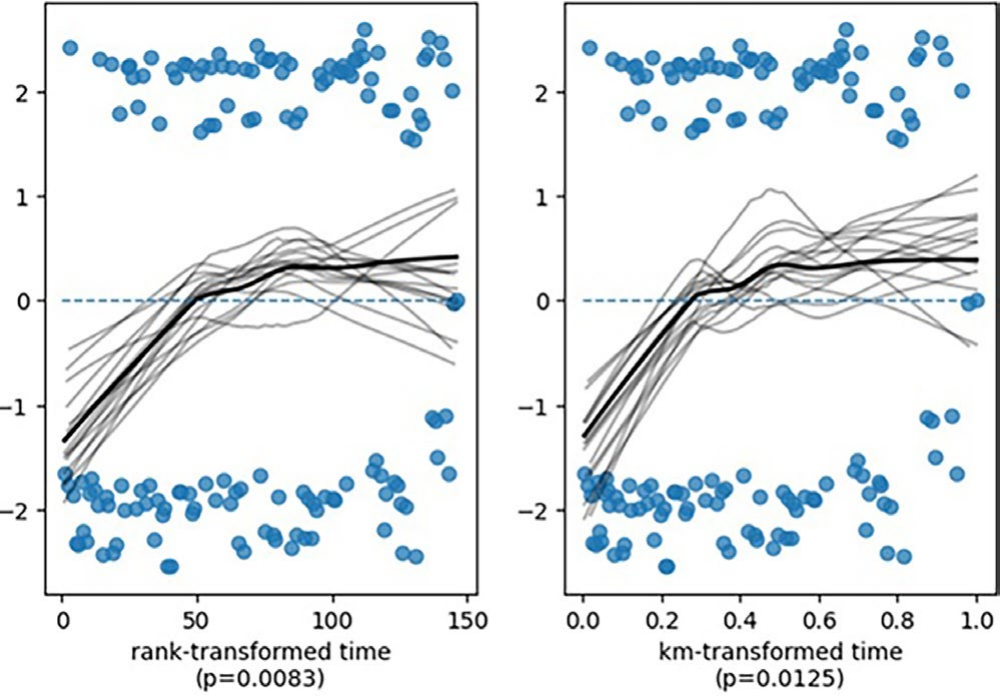
Overall survival (OS) stratified by pre‐AZA serum ferritin levels. Kaplan–Meier survival curves illustrating OS stratified by pre‐treatment serum ferritin levels. The analysis compares survival outcomes for patients with SF levels < 500 ng/mL, 500–1000 ng/mL and > 1000 ng/mL, demonstrating the negative impact of elevated SF on survival (*p* = 0.0012).

Median OS was significantly shorter in patients with SF levels > 1000 ng/mL compared to those with lower SF levels. The median OS for the < 500 ng/mL, 500–1000 ng/mL and > 1000 ng/mL groups was 18.2 months, 20.5 months and 10.1 months, respectively (*p* = 0.0012) (Figure 2). Kaplan–Meier survival analysis revealed a significantly reduced OS for patients with SF levels > 1000 ng/mL compared to those with SF levels < 500 ng/mL (log‐rank test *p* = 0.0023). Multivariate analysis confirmed that SF > 1000 ng/mL was an independent predictor of poor OS (adjusted HR: 2.15, 95% CI: 1.36–3.41, *p* = 0.0012). However, due to the violation of the proportional hazards assumption for ferritin (*p* = 0.0083), a stratified Cox model was used to ensure appropriate model fitting. While most covariates satisfied the assumption (*p* > 0.05), ferritin levels violated proportionality (*p* = 0.0083), indicating non‐constant effects over time (Figure 3). To account for this, a stratified Cox model was applied, adjusting for ferritin as a categorical variable (≤ 500 ng/mL, 500–1000 ng/mL, > 1000 ng/mL).

### 3.5 Subgroup Analysis and Independent Impact

**FIGURE 4:**
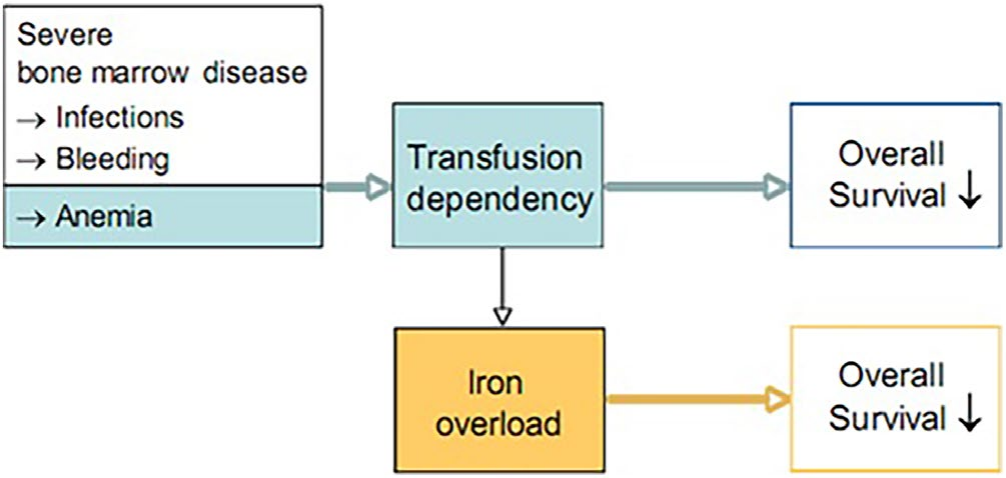
Independent impact of transfusion dependency and iron overload on survival in patients with myelodysplastic syndromes (MDS). The independent effect of transfusion dependency and iron overload on overall survival in patients with MDS. The figure highlights the combined impact of transfusion dependency and elevated SF levels in reducing survival outcomes in this patient population [16].

Subgroup analyses provided additional insights into the effect of transfusion dependence and IPSS risk categories on patient outcomes. Patients who were transfusion dependent at baseline exhibited poorer response rates compared to those who were transfusion independent (Table S1). Furthermore, patients with intermediate‐2 or high IPSS risk had a lower response rate and reduced OS compared to those with lower risk profiles. Figure 4 illustrates the independent impact of transfusion dependence and IO on survival in patients with MDS [16].

## 4 Discussion

The present study confirms that elevated pre‐treatment SF levels—an established surrogate for IO—are independently associated with lower OR and shorter OS in patients with MDS or AML treated with AZA. These findings remain significant even after adjusting for transfusion dependency, age and IPSS risk, reinforcing SF as a strong prognostic biomarker in this setting [3, 7, 17]. While SF can be influenced by inflammation, our data support the hypothesis that IO itself plays a major role in the observed poorer outcomes. Notably, patients with SF > 1000 ng/mL had markedly inferior response and survival, highlighting the clinical relevance of pre‐treatment iron status when considering hypomethylating agents. Moreover, the violation of the proportional hazards assumption for SF suggests its prognostic impact may be especially pronounced during early treatment phases. Transfusion dependency, although essential and often life‐sustaining in MDS management, contributes to cumulative IO and was also associated with adverse outcomes in our cohort. This aligns with prior studies showing that both transfusion burden and elevated ferritin levels negatively influence prognosis [5, 14]. However, our multivariate analysis suggests that ferritin captures additional prognostic information beyond transfusion status alone, particularly regarding long‐term iron accumulation and biological sequelae [3]. Notably, while previous studies such as Itzykson et al. [5] have identified transfusion dependence and cytogenetic risk as prognostic markers in AZA‐treated patients, ferritin was not evaluated as an independent variable in their multivariate models. Our study builds on this foundation by directly assessing pre‐treatment SF and demonstrating its independent association with both response and survival after adjusting for transfusion burden and IPSS score. Similarly, although Laribi et al. [6] reported that elevated ferritin levels were associated with inferior survival in high‐risk MDS patients treated with AZA, their conclusions were based on a small retrospective cohort (*n* = 48), with a notable proportion (17%) of early deaths prior to completing four cycles. Moreover, in that study, ferritin was not significantly associated with CRP or transfusion dependency in multivariate analysis, suggesting a more complex relationship with outcome. In contrast, our results in a larger real‐world population confirm the prognostic value of SF > 1000 ng/mL, supporting its inclusion in future risk stratification models and interventional trial design. Mechanistically, IO has been shown to generate oxidative stress, disrupt erythropoiesis and modulate epigenetic regulators of haematopoiesis [7, 18, 19]. These biological alterations may reduce the efficacy of AZA and contribute to disease progression. Consequently, there is growing interest in combining AZA with iron‐reducing interventions. Clinical data suggest that iron chelation may not only reduce SF levels but also improve haematological response and delay disease evolution [4, 16, 20]. Future trials should evaluate early chelation in patients with elevated SF, particularly prior to initiating hypomethylating therapy. We acknowledge limitations including the retrospective design and incomplete transfusion history. Furthermore, the lack of detailed information on prior transfusion burden and ESA use may limit the interpretation of SF as a purely iron‐related biomarker. Although none of the patients received ESAs before AZA due to their intermediate‐2 or high‐risk status, the retrospective nature of the study prevents full reconstruction of transfusional iron exposure. Nevertheless, SF levels > 1000 ng/mL likely reflect sustained IO and have consistently been linked to poor outcomes across studies. Moreover, SF may overestimate iron burden in inflammatory states [21]. Nevertheless, it is worth noting that ferritin levels exceeding 1000 ng/mL are unlikely to be solely explained by systemic inflammation, especially in the absence of acute infection or autoimmune disease. In the context of our cohort, these levels are more plausibly attributed to chronic transfusional IO and/or ineffective erythropoiesis. Additionally, comorbidities such as liver disease or metabolic syndrome may contribute to elevated ferritin levels independently of transfusional IO. These factors were not systematically recorded in our cohort and could not be controlled for, representing another limitation of our retrospective analysis. Thus, incorporating biomarkers such as hepcidin [22] or imaging modalities like MRI T2* may enhance IO assessment in future studies. These findings reinforce the need to proactively address transfusion‐related IO, particularly in low‐risk MDS patients with high transfusion demands. Even in the absence of a CR as defined by IWG criteria, early therapeutic intervention aimed at reducing transfusion intensity or achieving transfusion independence may prove beneficial. As patients progress to higher‐risk MDS, prior iron accumulation could negatively impact treatment response and overall prognosis. In conclusion, while RBC transfusions remain a cornerstone of supportive care in MDS, our findings underscore the importance of timely recognition and management of IO. Routine SF measurement prior to AZA initiation could improve risk stratification and identify patients who may benefit from iron‐reducing strategies. Integrating SF into prognostic models alongside IPSS and transfusion burden may enable more personalised treatment approaches and improved patient outcomes.

## Author Contributions

**Gloria Moreno Carrasco:** conceptualization (equal), investigation (lead), writing – original draft (equal), writing – review and editing (equal). **Rodolfo Matias Ortiz Flores:** conceptualization (equal), formal analysis (lead), validation (lead), visualization (lead), writing – original draft (equal), writing – review and editing (equal). **Regina García Delgado:** formal analysis (equal), investigation (equal), methodology (supporting), project administration (equal), resources (lead), supervision (equal), validation (equal), visualization (equal), writing – review and editing (supporting). **Borja Cidoncha Morcillo:** data curation (supporting), writing – review and editing (supporting). **Ana Isabel Rosell Mas:** conceptualization (equal), investigation (equal). **Dana Díaz Canales:** conceptualization (equal), investigation (supporting), writing – review and editing (supporting). **María Rodríguez González:** data curation (supporting), writing – review and editing (supporting). **Manuel Carrasco Gomariz:** conceptualization (supporting), investigation (supporting). **Alejandro Escamilla‐Sánchez:** formal analysis (equal), methodology (equal), project administration (equal), resources (equal), supervision (lead), validation (equal), visualization (equal), writing – review and editing (lead).

## Disclosure

The authors alone are responsible for the content and writing of this paper.

## Conflicts of Interest

The authors declare no conflicts of interest.

## Supporting information


**Table S1:** Response to treatment stratified by IPSS risk category (low, intermediate I, intermediate II, high).

## Data Availability

The data supporting the findings of this study are available from the corresponding author, Regina García Delgado, upon reasonable request. Data will be shared solely for non‐commercial purposes and in a manner that safeguards the confidentiality of participant information. Due to privacy and ethical restrictions, individual patient data are not publicly available.
