# Supplementary material for: Impact of Pre‐Treatment Serum Ferritin on Response and Survival in Myelodysplastic Syndromes Treated With Azacytidine: A Multivariate Analysis
Source: Cancer Med. 2025 Aug 4;14(15):e71127. doi: 10.1002/cam4.71127 (PMC12319231; doi:10.1002/cam4.71127)
Supplement: Supplementary file 1 — Table S1: Response to treatment stratified by IPSS risk category (low, intermediate I, intermediate II, high). [file CAM4-14-e71127-s001.docx]

Supplementary Table 1. Response to treatment stratified by IPSS risk category (low, intermediate I, intermediate II, high).

|  | IPSS Risk Category | Patients (n) | Overall Response (%) | Response at 4 months (%) | Response at 6 months (%) |  | P-values |
| --- | --- | --- | --- | --- | --- | --- | --- |
| 0 | Low | 46 | 62.5 | 58.3 | 70.8 |  | 0.0122 |
| 1 | Intermediate I | 92 | 48.9 | 46.7 | 70.6 |  | 0.4857 |
| 2 | Intermediate II | 55 | 45.4 | 50.9 | 74.5 |  | 1.0000 |
| 3 | High | 24 | 0.0 | 20.0 | 60.0 |  | < 0.0001 |

*Statistical comparisons between each IPSS risk category and the rest of the cohort were performed using Fisher’s exact test for overall response. Values represent two-sided p-values.*
